# Air volume not spray concentration determines in vivo efficacy of volatile organic compounds against *Plasmopara viticola*

**DOI:** 10.1038/s41598-026-40527-1

**Published:** 2026-02-16

**Authors:** Sabine Oberhofer, Sara Avesani, Michele Perazzolli, Peter Robatscher, Urban Spitaler

**Affiliations:** 1 Institute for Plant Health, Laimburg Research Centre, Laimburg 6, Auer (Ora), 39040 Italy; 2 Laboratory for Flavours and Metabolites, Laimburg Research Centre, Laimburg 6, Auer (Ora), 39040 Italy; 3 Center for Agriculture Food Environment (C3A), University of Trento, Via E. Mach 1, San Michele all’Adige, 38098 Italy; 4 Research and Innovation Centre, Fondazione Edmund Mach, Via E. Mach 1, San Michele all’Adige, 38098 Italy

**Keywords:** 2-phenylethanol, β-cyclocitral, linalool, Plasmopara viticola, volatile organic compounds, plant protection, Biotechnology, Microbiology, Plant sciences

## Abstract

Volatile organic compounds (VOCs) are produced by plants in response to abiotic and biotic stress stimuli. Two possible modes of action against plant pathogens have been reported for VOCs, such as the induction of plant resistance and direct inhibition of pathogen growth. The volatiles 2-phenylethanol, β-cyclocitral, and linalool exhibited inhibitory activity against downy mildew on grapevine leaf disks; however, their efficacy on whole plants remains unknown. The efficacy of the three VOCs against downy mildew was evaluated on potted grapevines under greenhouse conditions. Fumigation with VOCs in a limited air volume showed high efficacy for linalool and, to a lesser extent, for 2-phenylethanol and β-cyclocitral against *Plasmopara viticola* infections. However, VOCs showed no effect against *P. viticola* when applied as a liquid spray. In contrast, a standardized VOC dosage in a limited air volume led to a reduction in downy mildew severity, suggesting that improvements in application and formulation methods are required to allow long-lasting release and persistence of VOCs after application.

**Supplementary Information:**

The online version contains supplementary material available at 10.1038/s41598-026-40527-1.

## Introduction

In recent decades, many studies have been conducted to discover new active molecules for plant protection^1,2^, including the screening of biological control agents such as beneficial fungi or bacteria^3^ and natural substances such as plant and microbial extracts^4^. In particular, volatile organic compounds (VOCs) of plant and microbial origin are gaining interest as promising alternatives to synthetic fungicides, as their efficacy against diverse pathogens (e.g., *Rhizoctonia solani*,* Alternaria alternata*,* Fusarium oxysporum*,* Fusarium graminearum*) has been demonstrated^5–8^. Additionally, VOCs are associated with sustainable benefits, such as a reduced risk of residue persistence on plants and in the environment^9^, making them a promising new class of active substances for plant protection. However, the effect of VOCs against microbial pathogens was mainly tested in vitro and under laboratory conditions, while less information is available on their efficacy and application strategies on whole plants^10^.

VOCs comprise chemically diverse organic compounds characterized by high vapor pressure under ambient conditions^11^. They can be produced by bacteria, fungi, and plants^9^ and their biological activity has been investigated in numerous studies^12–14^. For example, plants emit VOCs in response to abiotic and biotic stimuli, such as attack by microbial pathogens, herbivory insects, mechanical damage, salinity, and drought^15^, as secondary metabolites through different metabolic pathways, including the plastidic methylerythritol phosphate pathway, cytosolic mevalonic acid pathway, shikimate pathway, phenylalanine pathway, and lipoxygenase pathway^11,16^. Plant VOCs play important roles in intra- and inter-kingdom communication^17,18^. In particular, VOCs produced by plants can inhibit the growth of phytopathogenic fungi, and two possible modes of action have been reported, namely the induction of plant resistance^19^ and the direct inhibition of pathogen growth^10,20,21^. For example, plant VOCs such as 2-phenylethanol, carvacrol, farnesene, and nonanal can directly inhibit the growth of plant pathogens^22–25^. Other VOCs, including β-cyclocitral, ionone, camphene, hexenal, isoprene, and pinene are known to induce defense-related processes against pathogens in different plant species^26–32^. Some plant VOCs, like caryophyllene, limonene, and linalool, have been shown to act through both mechanisms^22,24,28,33,34^.

Grapevine downy mildew, caused by the oomycete pathogen *Plasmopara viticola*, is a widespread disease in viticulture worldwide that causes considerable damage to grapevines^35,36^. The pathogen is endemic on wild *Vitis* species of North America and was first introduced in Europe at the end of the 19th century and has subsequently spread to Western and Eastern European wine-growing regions^37^. Effective control of the pathogen remained elusive until the introduction of the Bordeaux mixture in 1885^38^. Infections can affect all green tissues, including leaves, inflorescences, fruit clusters, and young bunches, leading to reduced photosynthetic assimilation and, consequently, substantial yield and quality losses^39^. The pathogen penetrates host tissues through stomata, with young leaves representing the most susceptible organs^40^. Therefore, overall young leaves are suitable for assessing plant protection measures. On leaves, initial symptoms manifest as yellow lesions on the upper surface, while inflorescences may turn brown. Leaf lesions are often oily and angular and later become necrotic and brown as they age^35^. Under conditions of high humidity, new sporulation occurs on the abaxial leaf surface, with sporangia emerging through stomata^41^. Each sporangium releases four to eight biflagellate zoospores that swim through surface water to the stomata, where they initiate new secondary infections^42^.

Control strategies against *P. viticola* in viticulture include the application of chemical pesticides, such as multi-site active substances like phthalimides and dithianon, or single-site active substances like quinone outside inhibitors, carboxylic acid amides, benzamides, and phenylamides^43^. Furthermore, phosphonates, which act primarily as host defense inducers, are also synthetic compounds and therefore classified as chemical pesticides^44^. Additionally, copper-based formulations are multi-site fungicides permitted in organic farming. However, they have the disadvantage to accumulate in the soil, leading to heavy metal contamination^45,46^. Although chemical control strategies are the most effective in controlling the pathogen, target-site fungicides carry the risk of selecting resistance traits in *P. viticola* populations^47^. For example, *P. viticola* can develop resistance to quinone outside inhibitors^48,49^, phenylamides^50^, and carboxylic acid amides^51,52^, all of which are important active compounds in numerous plant protection products^43^. These resistances cause further difficulties in the control of the pathogen. Moreover, numerous active substances are currently under scrutiny for substitution in Europe^53^. The use of copper is limited to a maximum of 28 kg ha^− 1^ over seven years^54^, and further limitations are expected^55,56^. Since effective control of *P. viticola* in viticulture is becoming increasingly challenging, alternative strategies are highly needed to counter infection pressure.

In grapevine, the VOCs 2-phenylethanol, β-cyclocitral, and linalool are mainly produced by *P. viticola*-resistant genotypes, such as Bianca^57,58^, Croatian cultivars^59^, Kober 5BB, SO4, Solaris^60^, and a pyramided genotype^61^, indicating a potential role of VOCs in resistance to *P. viticola*. Laboratory application of volatile 2-phenylethanol, β-cyclocitral, and linalool reduced downy mildew symptoms on grapevine leaf disks in vitro^33,60,62^, indicating that these VOCs could serve as promising agents for grapevine protection.

This study aimed to assess the efficacy of volatile 2-phenylethanol, β-cyclocitral, and linalool against *P. viticola* on potted grapevines under greenhouse conditions. Furthermore, fumigation in a limited air volume and standard liquid applications of the three VOCs were compared to understand the relevance of the application method.

## Materials and methods

### Volatile organic compounds (VOCs)

The three VOCs 2-phenylethanol (2-phenylethan-1-ol), β-cyclocitral (2,6,6-trimethylcyclohex-1-ene-1-carbaldehyde), and linalool ((±)-3,7-dimethylocta-1,6-dien-3-ol) (Sigma-Aldrich, Merck) were used as active substances in this study (Table 1), based on their demonstrated efficacy against *P. viticola* on grapevine leaf disks observed in vitro^33,60,62^. It was reported that the three selected VOCs are significantly more abundant in resistant grapevine genotypes (BC4, Kober, 5BB, SO4, and Solaris) than in the susceptible genotype (*Pinot noir*) following an infection with *P. viticola*^60^.

**Table 1:** Chemical characteristics of the three VOCs used for fumigation in a limited air volume and for as a liquid spray application on grapevines.

VOC	Abbreviation	CAS number	Molecular formula	Molar weight (g moL^− 1^)	Density (mg µL^− 1^)	Purity (%)
2-Phenylethanol	2-PE	60-12-8	C_8_H_10_O	122.16	1.023	99.0
β-Cyclocitral	β-CC	432-25-7	C_10_H_16_O	152.23	0.943	84.21
Linalool	LIN	78-70-6	C_10_H_18_O	154.25	0.87	97.0

### Plant material and maintenance of *Plasmopara viticola* inoculum

Grapevine plants (*V. vinifera* cultivar Vernatsch/Schiava, grafted on rootstock SO4) were potted into black 4.5-L containers (15 × 15 × 20 cm) filled with soil (acid peat with NPK fertilizer, Geotec S.r.l., Italy) and grown under greenhouse conditions (temperature: 20–24 °C; photoperiod: 16 h light; relative humidity 40%). Plants were irrigated weekly until at least five leaves were fully developed.

The *P. viticola* strain was collected from an untreated vineyard (*V. vinifera* cultivar Chardonnay) in the Trentino-Alto Adige region (northern Italy) in 2023 and maintained under greenhouse conditions through continuous reinoculation of fresh potted grapevines every three weeks. For long time storage, sporulated leaves were stored at -20 °C to maintain spore viability. Molecular identification of the used *P. viticola* strain was confirmed by DNA extraction from a sporangia suspension, followed by amplification and sequencing of the ITS1 gene region^63^. The obtained sequence was deposited in the NCBI GenBank (accession number: PQ526750).

### VOC fumigation in a limited air volume

**Fig. 1:**
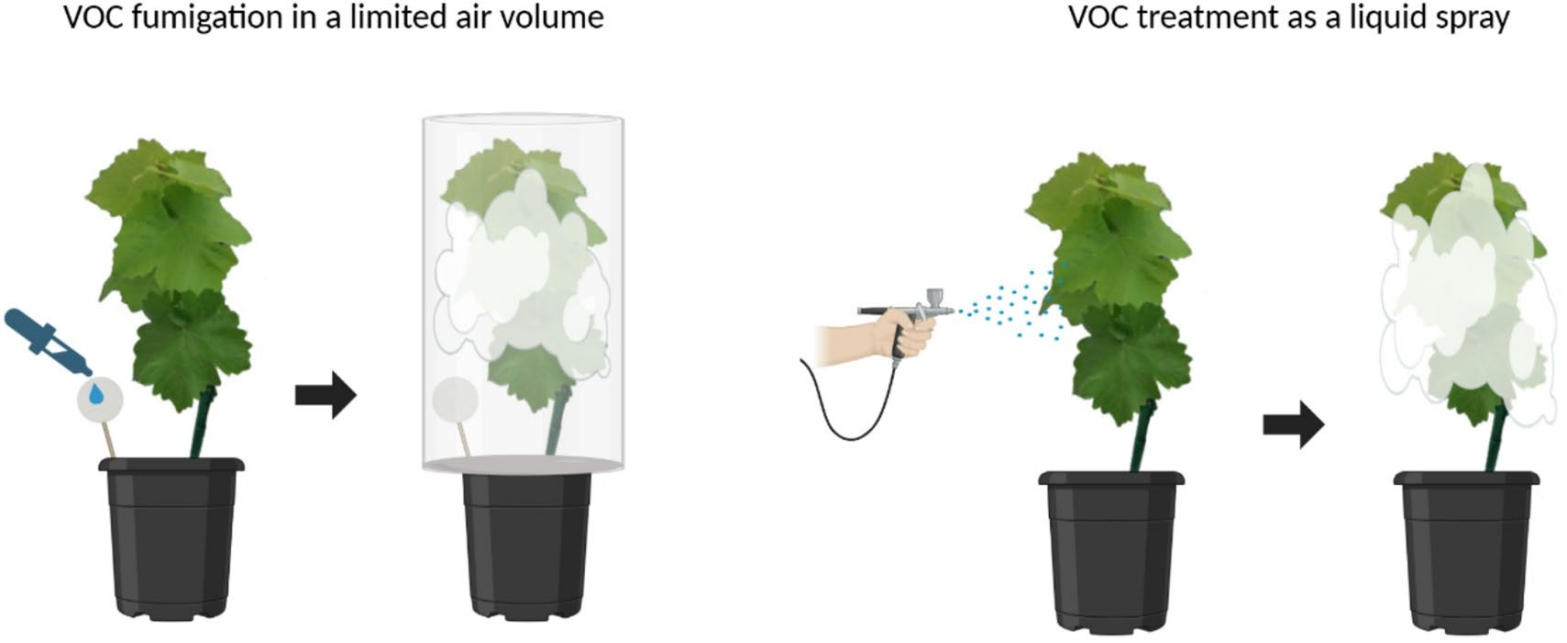
Graphical illustration of the VOC fumigation in a limited air volume and liquid spray application on potted grapevines under greenhouse conditions. For the fumigation treatment in a limited air volume, the VOC formulation was applied to filter paper, and the plant was immediately covered with a glass container. For the liquid spray application, the VOC formulation was applied directly to the leaf surfaces using an airbrush.

For the VOC fumigation treatment in a limited air volume, transparent glass containers (cylindrical jars: 38 cm height, 20 cm diameter) with a volume of 12 L were used to cover the aerial part of each grapevine plant after VOC application and during the evaporation process (Fig. 1). The amount of VOC required for each treatment was calculated based on the density and purity of the respective compound (Table 1) according to the following equation:

$$\:VOC\:amount\:\left[L\right]=\frac{\:Treatment\:concentration\:\left[{mg\:L}^{-1}\right]\times\:Volume\:of\:glass\:container\:\left[L\right]}{VOC\:density\:\left[{mg\:L}^{-1}\right]\times\:VOC\:purity\:\left[\%\right]}\times\:100$$

To increase solubility, double the amount of dimethyl sulfoxide (DMSO, CAS No. 67-68-5, Sigma-Aldrich, Merck) was added to the calculated amount of each VOC, and the suspension was then diluted tenfold in sterile deionized water, corresponding to a final concentration of 10% (v/v) of DMSO (Supplementary Table 1). Control plants were treated with the same amount of DMSO used for the highest VOC concentration. Accordingly, controls treatments consisted of DMSO and sterile deionized water without any volatile compound.

Prepared solutions of the three VOC treatments or the control were applied onto a filter paper (18 cm diameter; Macherey-Nagel, Germany) that was attached to a stick anchored in the potting soil. Immediately after application, each plant was covered with the glass container, and the lower edge of the glass container was sealed with a white polyethylene-plastic film to ensure VOC retention within the defined volume (Fig. 1). The glass containers remained on the plants overnight for 16 h to maintain VOC exposure around the grapevine.

In the first trials, dosage ranges of 2-phenylethanol (0.1–200 mg L⁻¹ air volume), β-cyclocitral (0.1–200 mg L⁻¹ air volume), and linalool (1–10 mg L⁻¹ air volume) were tested to determine the optimal, non-phytotoxic dose for each VOC (Table 2). Dosages were selected based on in vitro data from leaf disk assays^33,60,62^. Between six and nine single leaves per grapevine were used as replicates, depending on the plant size (*n* ≥ 6) in these experiments.

The concentration of each VOC for the final validation trial was 3 mg L^− 1^ air volume of 2-phenylethanol (corresponding to 36 µL on the filter paper), 15 mg L^− 1^ air volume of β-cyclocitral (corresponding to 227 µL on the filter paper), and 5 mg L^− 1^ air volume of linalool (corresponding to 71 µL on the filter paper), assuming complete evaporation of each VOC in the glass container. Five plants were treated with linalool and β-cyclocitral, and six plants were treated with 2-phenylethanol and the control treatment (DMSO and water). Six leaves per plant were validated. The VOCs linalool and 2-phenylethanol were tested simultaneously. All plants were arranged randomly. The mean infected leaf area was calculated per plant, and single plants served as replicates (*n* = 5–6).

### VOC application as a liquid spray

VOC application as a liquid spray was performed using an airbrush (Fengda, Germany) operated at 2 bar pressure (Fig. 1). Each treatment (10 mL) was sprayed evenly onto both the adaxial and abaxial surfaces of grapevine leaves of a single plant. The spray suspensions were prepared as follows: the calculated amount of VOC was pipetted into a 20 mL tube (LLG Labware, Germany), diluted with double the amount of DSMO, and the adjusted to a final volume of 10 mL with sterile deionized water (Supplementary Table 1). Control plants were sprayed with the same amount of DMSO as that used in the highest concentration of the VOC treatment.

In the first trials, different dosages of the VOCs (100, 500, 1,000, 1,500, 5,000, and 20,000 mg L^− 1^ spray volume) were applied to single plants, whereas control plants were treated with DMSO and water. Single leaves per plant served as replicates (*n* = 6) to assess the phytotoxic effects on the grapevines.

For the final validation trial, the intermediate dosage of 15,000 mg L^− 1^ in spray volume was applied to each plant for the three VOCs. At this concentration, phytotoxic damage was still within acceptable limits, whereas a concentration of 20,000 mg L^− 1^ caused strong phytotoxic damage. Three plants were independently treated for the final trial, and six leaves per plant were evaluated for *P. viticola* infection (*n* = 18). All plants were arranged randomly.

### Inoculation with *Plasmopara viticola*

All VOC-treated plants and control plants were inoculated with *P. viticola* 24 h after fumigation or spray application. To obtain the *P. viticola* inoculum, grapevine leaves showing disease symptoms were incubated in a closed plastic box (60 × 40 × 11 cm; Giganplast, Italy) on a moistened paper with the abaxial leaf surface upwards. The leaf surfaces were sprayed with sterile deionized water, and boxes were incubated for 16 h in the dark at 95 ± 5% RH to promote pathogen sporulation. Sporangia were collected by washing the abaxial leaf surfaces bearing freshly sporulating lesions with cold (4 °C) sterile deionized water. The inoculum concentration was then adjusted to 1.0 × 10^5^ sporangia mL^− 1^ using a hemocytometer under a light microscope, as previously described^21^. Plants were moistened with sterile deionized water and immediately inoculated with 20 mL of a freshly prepared *P. viticola* sporangial suspension using a hand-held spray bottle. Plants were covered for 24 h with the glass container, internally moistened with sterile deionized water to maintain 100% humidity. Plants were then randomly placed under greenhouse conditions. Maintaining relative humidity of 40% in the greenhouse effectively inhibited *P. viticola* sporulation and prevented cross-contamination among plants during incubation.

### Assessment of diseased leaf area and phytotoxic effects

Six days after inoculation with *P. viticola*, the diseased leaf area (DLA) was evaluated for all treatments and the efficacy was calculated. Six leaves from VOC-treated and control plants were collected without petioles and placed in plastic boxes on a layer of moistened paper, with the abaxial surface facing upwards. To promote sporulation, the leaves were sprayed with sterile deionized water and incubated overnight in the closed plastic box under high humidity at 21 °C. After 24 h, each leaf was photographed against a black background using a camera (Canon EOS 300D) mounted on a tripod. The DLA in percentage was quantified using the software Fiji (version 2.17.0)^64^ by color threshold segmentation to distinguish between sporulating and total leaf area^65^(Supplementary Fig. 1).

Treatment efficacy (%) was calculated according to the following equation:

$$\:Treatment\:efficacy=\:\frac{(DLA\:of\:control\:treatment-\:DLA\:of\:VOC\:treatment)}{DLA\:of\:control\:treatment}\times\:100$$

Potential phytotoxic effects of VOC treatments were visually assessed by evaluating the presence and severity of necrotic symptoms. Necrosis severity was scored on a five-point scale from 0 to 4, where 0 = 0% leaf area with necrotic spots; 1 = 1–20% leaf area with necrotic spots; 2 = 20–50% leaf area with necrotic spots; 3 = 60–90% leaf area with necrotic spots; and 4 = > 90% leaf area with necrotic spots.

### Statistical analysis

Data were analyzed with R (version 4.3.2) and RStudio (version 2023.09.1). The data were first tested for normal distribution using the Shapiro-Wilk test. To determine significant differences (*p* < 0.05) between fumigation in limited air volume and the corresponding control, one-way ANOVA followed by a Tukey’s post-hoc test was performed (normal distribution: Shapiro-Wilk test *p* > 0.05; variance homogeneity: Levene test *p* > 0.05). For treatments applied as a liquid spray, significant differences (*p* < 0.05) were tested using the Kruskal-Wallis rank sum test with Dunn’s post hoc test, since data were not normally distributed (normal distribution: Shapiro-Wilk test *p* ≤ 0.05; variance homogeneity: Levene test *p* ≤ 0.05).

## Results

### Effects of VOC fumigation in a limited air volume

Trials were conducted to determine the optimal dosage for VOC fumigation in a limited air volume against *P. viticola*, ensuring that no phytotoxic occurred on the plants (Table 2). Fumigation in a limited air volume achieved reduction in *P. viticola* severity exceeding 30% at the following dosages: 2-phenylethanol at 2 mg L⁻¹ resulted in a 32.3% reduction, β-cyclocitral at 5 mg L⁻¹ achieved a 39.6% reduction, and linalool at 4 mg L⁻¹ reduced severity by 42.5%.

**Fig. 2:**
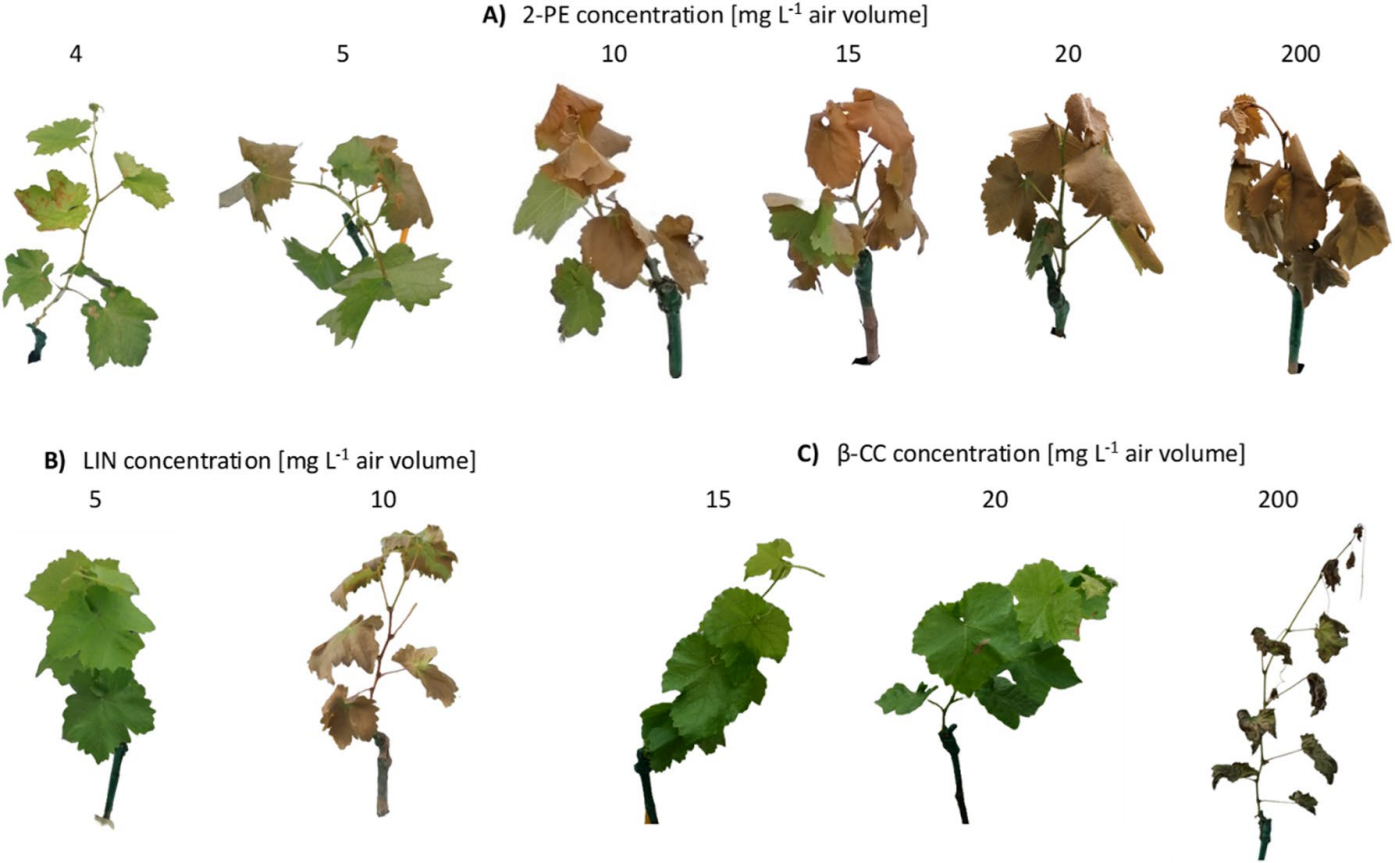
Phytotoxic effects on potted grapevines following fumigation in a limited air volume. Potted grapevines were treated with different dosages of (**A**) 2-phenylethanol (2-PE), (**B**) linalool (LIN), and (**C**) β-cyclocitral (β-CC). Photos were taken 24 h after treatment.

A dosage of 2-phenylethanol of 4 mg L^− 1^ caused necrotic spots of up to 20% of the leaf surface (phytotoxic level 1), while 5 mg L^− 1^ resulted in necroses covering 50% of the leaf area (phytotoxic level 2) (Fig. 2A). For both 2-phenylethanol and linalool, a dosage of 10 mg L^− 1^ in air volume caused more than 90% leaf necrosis (phytotoxic level 4) and death of the shoots 24 h after treatment (Fig. 2A, B). No visible damage (phytotoxic level 0) was observed for β-cyclocitral at 15 mg L^− 1^ in air volume, whereas up to 20% of leaf area showed necrotic spots (phytotoxic level 1) at 20 mg L^− 1^ in air volume (Fig. 2C).

**Table 2:** Tested dosages of the volatile 2-phenylethanol (2-PE), β-cyclocitral (β-CC), and linalool (LIN) in a limited air volume to evaluate efficacy against *P. viticola*. The efficacy of each treatment was calculated relative to the DMSO and water treated control (± SD). Phytotoxic damage on leaves is described by severity levels as follows: 0 = 0% leaf area with necrotic spots; 1 = 1–20%; 2 = 20–50%; 3 = 60–90%; and 4 = > 90% leaf area with necrotic spots.

VOC	Concentration in a limited air volume [mg L^− 1^ air volume]	Efficacy against* Plasmopara viticola*	Phytotoxic level
2-PE	0.1	No effect	0
	1	9.1% ± 27.3	0
	2	32.3% ± 0.2	0
	3	60.8% ±20.8	0
	4	63.9% ± 35.7	1
	5	–	2
	10	–	4
	15	–	4
	20	–	4
	200	–	4
β-CC	0.1	No effect	0
	1	No effect	0
	2	No effect	0
	3	No effect	0
	4	No effect	0
	5	39.6% ± 22.2	0
	10	27.8% ± 33.2	0
	15	55.9% ± 13.8	0
	20	42.5% ± 30.7	1
	200	–	4
LIN	1	No effect	0
	2	No effect	0
	3	No effect	0
	4	42.5% ±28.7	0
	5	97.8% ±0.86	0
	10	–	4

**Fig. 3:**
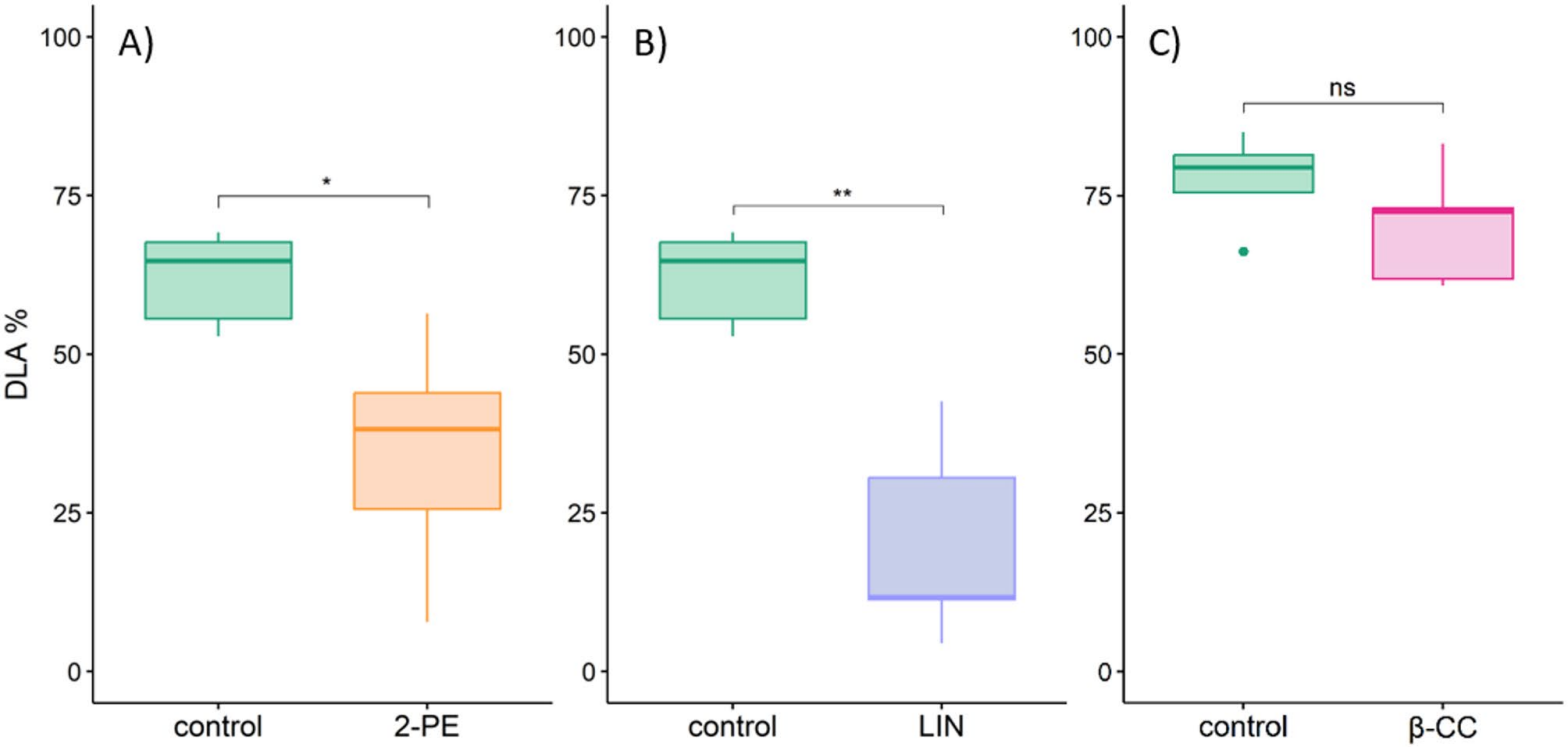
Effect of fumigation in a limited air volume with (**A**) 3 mg L^− 1^ 2-phenylethanol (2-PE), (**B**) 5 mg L^− 1^ linalool (LIN), and (**C**) 15 mg L^− 1^ β-cyclocitral (β-CC) on diseased leaf area (DLA) six days after inoculation with *P. viticola* (*n* = 5–6). Control plants were fumigated with DMSO and water. Asterisks indicate statistically significant differences (*p* < 0.05) compared with the respective control according to Tukey’s post-hoc test (*0.01, **0.001).

Based on phytotoxicity assessment and efficacy against *P. viticola*, dosages that achieved maximal disease reduction without visible phytotoxic effects were selected for further trials on potted grapevines: 3 mg L^− 1^ in air volume for 2-phenylethanol, 15 mg L^− 1^ for β-cyclocitral, and 5 mg L^− 1^ for linalool. At these selected dosages, linalool and 2-phenylethanol significantly reduced the DLA compared to the control plants (F_2_ = 12.83, *p* < 0.001), with mean efficacies of 67.6 ± 25.5% and 44.2 ± 25.4%, respectively (Fig. 3A, B). In contrast, no significant reduction in DLA was observed in β-cyclocitral-treated plants (F_1_ = 1.927, *p* = 0.203) (Fig. 3C).

### Effects of VOC application as a liquid spray

**Fig. 4:**
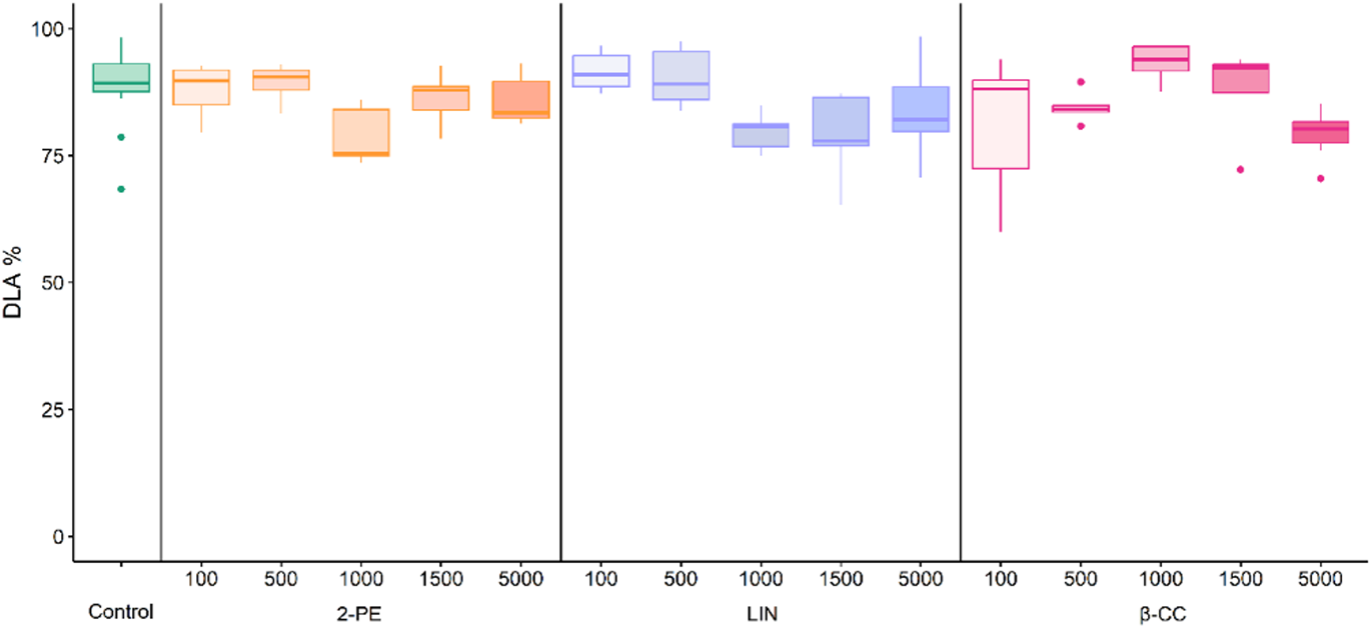
Effect of liquid spray application with different dosages (100, 500, 1,000, 1,500, and 5,000 mg L^− 1^) of 2-phenylethanol (2-PE), linalool (LIN) and β-cyclocitral (β -CC) on diseased leaf area (DLA) six days after inoculation with *P. viticola* (*n* = 5). Control plants were treated with deionized. No significant differences (*p* > 0.05) between VOC-treated and control plants were found according to Kruskal-Wallis rank sum test with Dunn’s post hoc test.

Application of 2-phenyethanol, β-cyclocitral, and linalool as a liquid spray did not reduce *P. viticola* symptoms at any of the tested dosages (100, 500, 1,000, 1,500, and 5,000 mg L^− 1^) (Fig. 4). Moreover, no phytotoxic damage (0% leaf area with necrotic spots) was observed on grapevine leaves treated with volatile 2-phenylethanol, β-cyclocitral, and linalool up to a dosage of 5,000 mg L^− 1^ in liquid spray volume. At a dosage of 20,000 mg L^− 1^ phytotoxic damage occurred, with over 90% of leaf area showing necrotic spots (phytotoxic level 4) for all three VOCs, making the inoculation with *P. viticola* and DLA evaluation impractical.

**Fig. 5:**
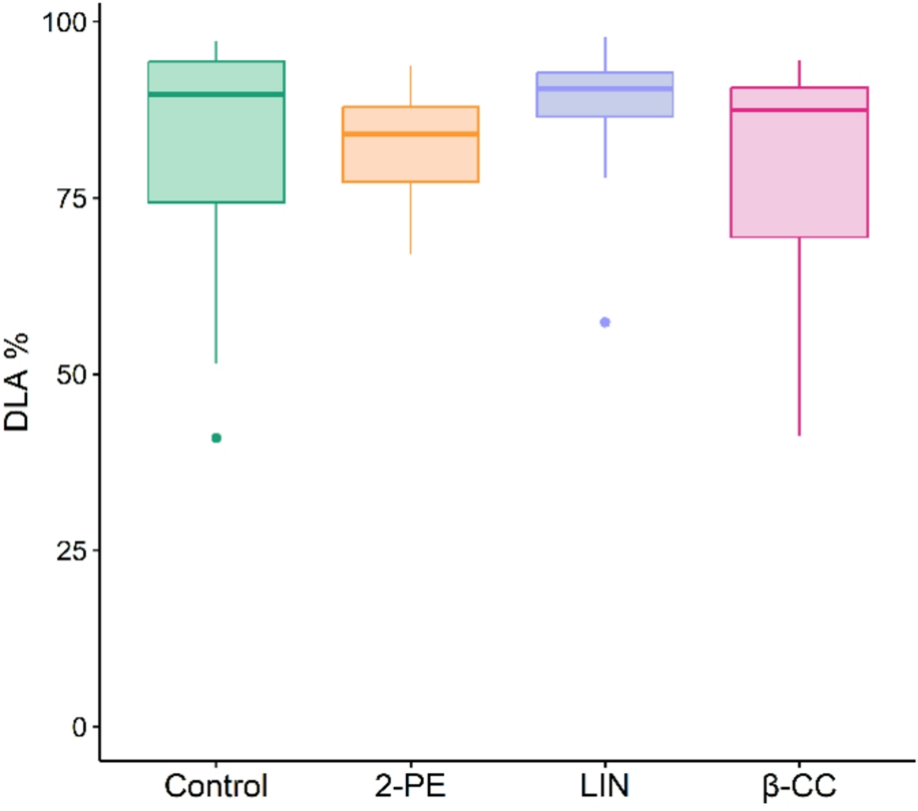
Effect of liquid spray application with 15,000 mg L^− 1^ in liquid spray volume of 2-phenylethanol (2-PE), linalool (LIN) and β-cyclocitral (β-CC) on diseased leaf area (DLA) six days after inoculation with *P. viticola* (*n* = 18). Control plants were treated with DMSO and water. No significant differences (*p* > 0.05) between VOC-treated and control plants were found according to Kruskal-Wallis rank sum test with Dunn’s post hoc test.

**Fig. 6:**
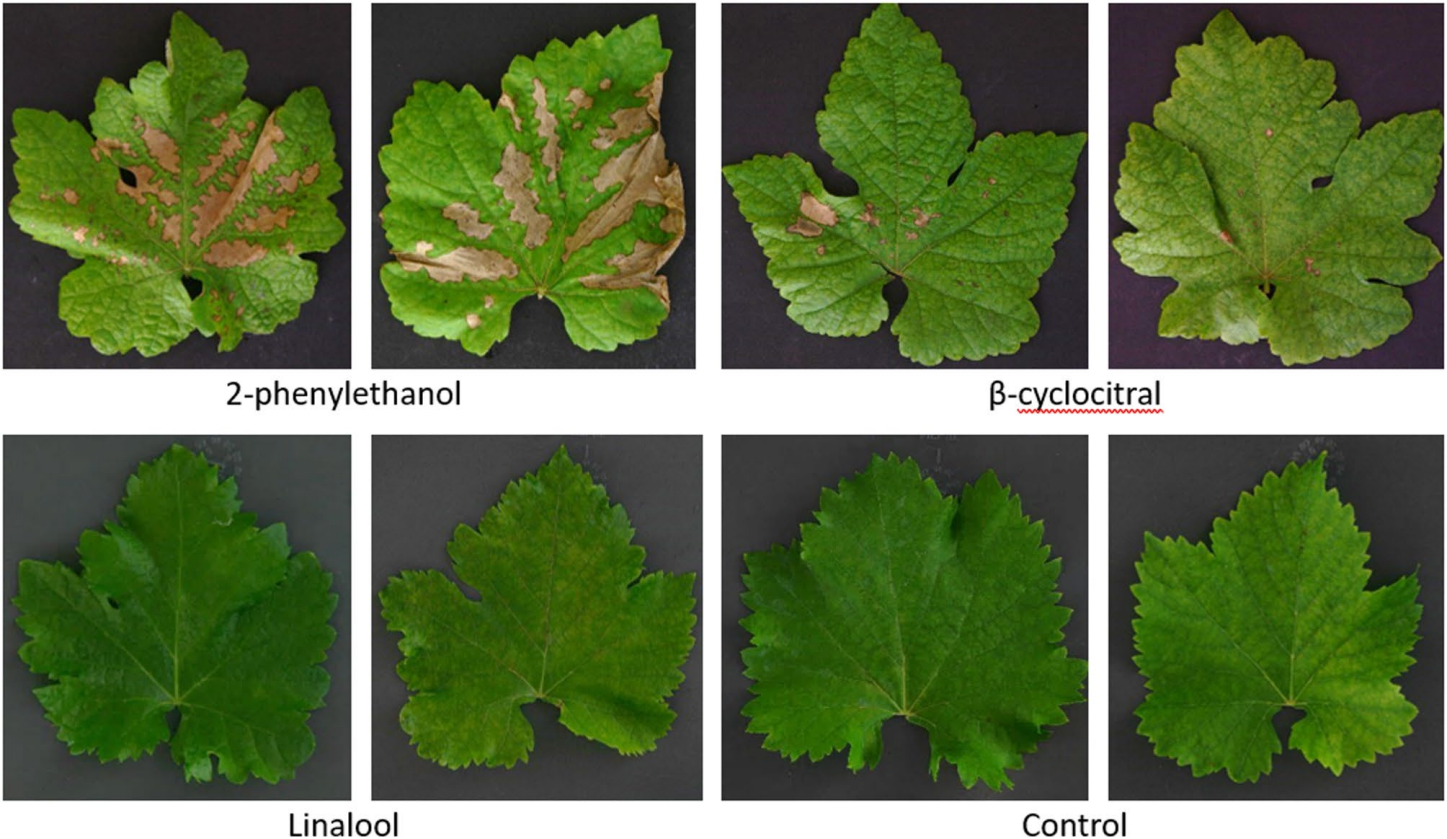
Phytotoxic damage of VOC application as a liquid spray at 15,000 mg L^− 1^ of 2-phenylethanol 15,000 mg L^− 1^ β-cyclocitral, or 15,000 mg L^− 1^ linalool. Treatment with β-cyclocitral caused small necrotic spots on grapevine leaves, 2-phenylethanol caused severe necrotic spots, whereas no phytotoxic effects were observed for linalool and the DMSO and water treated control.

In the final trial, application with 15,000 mg L^− 1^ VOCs in liquid spray volume confirmed no significant effect on the DLA of treated plants (Kruskal-Wallis χ^2^ = 6.7846, df = 3, p-value = 0.079) (Fig. 5). A concentration of 15,000 mg L^− 1^ in liquid spray volume was the highest amount applicable, where an inoculation with *P. viticola* was still possible. At thedosage of 15,000 mg L^− 1^ in liquid spray volume, slight phytotoxic effects were observed: up to 50% of the leaf area for 2-phenylethanol (phytotoxic level 2) and for up to 20% of the leaf area for β-cyclocitral (phytotoxic level 1). Linalool caused no phytotoxicity (phytotoxic level 0) at a concentration of 15,000 mg L^− 1^ in spray volume (Fig. 6).

## Discussion

VOCs represent a class of active substances that may also be of interest for the use as plant protection products^9^. While most previous research has focused on their effects on pathogens in vitro, such as on growth media or leaf disks, little is known about their efficacy when applied to potted plants^24,60^. The effects of 2-phenylethanol, β-cyclocitral, and linalool against *P. viticola* have previously been shown on susceptible grapevine leaf disks, showing good efficacy in reducing the infected leaf area in dish chambers when applied in air volume at dosages of 10 mg L^− 1^ for linalool and 20 mg L^− 1^ for 2-phenylethanol and β-cyclocitral^33,60,62^.

In the present study, these VOCs were applied to potted grapevines under greenhouse conditions to evaluate their efficacy against *P. viticola* and to explore different application methods. Our results show that fumigation in limited air volume can reduce disease severity at lower dosages than those previously required in vitro, highlighting the responsiveness of whole plants, whereas liquid spray applications were largely ineffective. These findings emphasize both the promise and the challenges of using VOCs for plant protection, particularly the need for formulations or application strategies that prolong contact with the plant and reduce rapid volatilization.

To achieve a defined air volume, potted grapevines were placed under glass containers to retain the evaporating substances around the plant. Additionally, VOCs spraying was conducted to mimic the standard application method of plant protection products under field conditions^66^.

Fumigation in a limited air volume revealed similar results for linalool and 2-phenylethanol, demonstrating the ability to decrease *P. viticola* symptoms. The optimized dosages for fumigation in a limited air volume without phytotoxic damage were lower for the in vivo application on potted plants (3 mg L^− 1^ in air volume for 2-phenylethanol and 5 mg L^− 1^ in air volume for linalool) compared to those required in vitro on leaf disks^33,60,62^, suggesting high responsiveness of potted plants. The antimicrobial activity of linalool has been reported against other plant pathogens, such as *Colletotrichum lindemuthianum* in common bean^24^, *Botrytis cinerea* in strawberry and tomato^67,68^ and *Fusarium oxysporum* in tomato^69^. Likewise, 2-phenylethanol has shown antimicrobial activity against diverse phytopathogens, including *Aspergillus flavus* in vitro^70^, *Botrytis cinerea* in strawberry^71^, and *Fusarium graminearum* in wheat^72^. These findings support the effectiveness of both substances as potential active agents for protecting grapevines from *P. viticola* and suggest possible side effects on other grapevine-associated microorganisms, such as grapevine crown gall (*Agrobacterium tumefaciens*)^73^, or grey mould (*Botrytis cinerea*)^74^. It has been shown that pure individual VOCs of diverse chemical structures can affect further organisms such as phytopathogenic bacteria *Agrobacterium tumefaciens*, the *Arabidopsis thaliana* plant, and the fruit fly *Drosophila melanogaster*, across distant taxonomic groups sharing the same ecological niche^75^. For example, altered VOC emissions can influence the oviposition behaviour and attraction of the grapevine moth *Lobesia botrana*^76^, with a binary blend of acetic acid and 2-phenylethanol significantly attracting both male and female moths^77^. In contrast, linalool significantly deters oviposition in Mediterranean fruit flies *Ceratitis capitata*^78^. These findings indicate that potential side effects on non-target organisms should be carefully investigated in future studies.

In contrast, β-cyclocitral showed only minor efficacy against *P. viticola* during fumigation in a limited air volume, although its inhibitory effect against *P. viticola* was previously observed on grapevine leaf disks^60^. In previous studies, 20 mg L^− 1^ of β-cyclocitral in air volume significantly reduced the disease severity compared to the control^60,62^. When testing different concentrations of β-cyclocitral to assess the phytotoxic effects, a reduction in the infected leaf area was also observed. Based on the low efficacy of β-cyclocitral in this study for fumigation in a limited air volume, it can be concluded that this compound is less suitable for the control of *P. viticola*. However, possible synergistic effects with other VOCs could enhance its efficacy, as demonstrated for VOCs from powdery mildew *Oidium* sp. such as 2-phenylethyl alcohol, propanoic acid, 2-methyl-, methyl ester, propanoic acid, 2-methyl-, ethyl ester against phytopathogenic oomycete *Pythium ultimum*^79^. Similarly, dual exposure to the green leaf volatile (*Z*)-3‐hexenyl acetate and the aromatic volatile indole increased plant resistance against caterpillars compared to single volatiles^80^. Furthermore, β-cyclocitral produced by the cyanobacteria *Microcystis* sp. has shown efficacy in other pathosystems, for example against the green algae *Chlorella pyrenoidosa*^81^ and *Chlamydomonas reinhardtii*^82^. Thuse, possible additive effects of β-cyclocitral with other active ingredients should be investigated in further studies.

Application of VOCs as liquid spray did not reduce *P. viticola* infection on potted grapevines for any of the three compounds at different dosages. Spraying techniques are commonly used to apply active compounds in the field for crop protection^66^. These results therefore provide new insights for further development and use of such compounds in plant protection. A typical characteristic of VOCs is the high vapour pressure under environmental conditions^11^, as well as high diffusivity in air and water^10^. In the conducted experiments, spraying the three VOCs directly onto plants may have led to rapid volatilization and disappearance within the greenhouse, whereas during fumigation in a limited air volume, the active substances were retained around the plants for 24 h due to the glass containers. This could also explain why covered plants exhibit stronger phytotoxic reactions, even at lower VOC concentrations.

These findings indicate that the duration of contact between plants and VOCs plays a crucial role in determining their efficacy as antimicrobial compounds or inducers of plant resistance. The necessity of long-term exposure for achieving beneficial effects has also been demonstrated in other studies, for example, in promoting plant growth in *Arabidopsis*^83^. In a previous study, volatile exposure for 6 h did not reduce infection with *Pseudomonas syringae*, whereas increased exposure over 24 h at the same dosage significantly enhanced plant resistance^84^. Based on the present results for both fumigation and spray treatments, it can be concluded that conventional application techniques are inadequate for VOCs used without co-formulants. New application methods or compound formulations should therefore be developed. Such an approach could include the addition of persistent substances to allow prolonged fumigation and persistence of VOCs after application, thereby enabling sustained contact with the plant. For example, bead or matrix formulations can continuously release volatiles^85^. The alarm pheromones (*E*)-β-farnesene and (*E*)-β-caryophyllene, when formulated in alginate gel beads, achieved slow release of active molecules for over 15 days under field conditions^86^. Moreover, microencapsulation techniques can be used to control the volatility and release properties of VOCs or essential oils^87,88^. Such microcapsules can be applied to plant surfaces using conventional pesticide sprayers^85^, facilitating the practical application of VOCs under field conditions. Another potential approach is plant soaking in VOC formulations, as drenching plants with formulations containing VOCs has been shown to reduce disease incidence caused by *Pseudomonas syringae* pv. *lachrymans*^89^ or naturally occurring viruses^90^ under field conditions. In addition, intercropping systems represent a promising strategy to sustain emitted VOCs by those plants in the field over extended periods^91^. Recently, intercropping grapevines with hoary stock (*Matthiola incana*) was shown to effectively reduce *P. viticola* incidence through antimicrobial VOC emissions from hoary stock^92^. Nevertheless, the risk of phytotoxicity should be considered in further assessments of VOCs as active substances for plant protection. As demonstrated in this study, VOCs can cause major plant damage with even small changes in dosage. Phytotoxic effects have also been reported for in vitro treatments with linalool at 25 mg L^− 1^ air volume^33^ and for VOC treatments on such as apricot, peach, and nectarine using *trans*-2-hexenal^23^. The phytotoxicity of VOC-based disease management is largely dose- and exposure-dependent, as shown in this study. However, such risks could be minimized in the vineyard through controlled-release formulations^66,86,88^, natural emission strategies to avoid direct foliar contact^92^, and the use of synergistic VOC blends that maintain efficacy at low concentrations^77^. Additionally, the timing of VOC exposure can decrease phytotoxic damage on sensitive tissues, like young leaves or flowers^93^.

In conclusion, VOCs have the potential to contribute to more sustainable plant protection, as their antimicrobial effects and their ability to activate plant defence mechanisms have been demonstrated in several studies. However, their volatile properties pose major challenges for developing suitable formulations for agricultural use. Further research is required to enable practical application of these compounds under field conditions. In particular, the development of appropriate formulations that prevent rapid VOCs evaporating and allow prolonged contact with the plant could substantially improve their efficacy.

## Supplementary Information

Below is the link to the electronic supplementary material.

Supplementary Material 1

Supplementary Material 2

## Data Availability

The data that support the findings of this study are openly available in https://zenodo.org/ .
