# Supplementary material for: Air volume not spray concentration determines in vivo efficacy of volatile organic compounds against Plasmopara viticola
Source: Sci Rep. 2026 Feb 16;16:9325. doi: 10.1038/s41598-026-40527-1 (PMC13000241; doi:10.1038/s41598-026-40527-1)
Supplement: Supplementary file 1 — Supplementary Material 1 [file 41598_2026_40527_MOESM1_ESM.docx]

**Supplementary Table 1:** Tested dosages and applied amounts of the volatile organic compounds (VOCs) 2-phenylethanol (2-PE), β-cyclocitral (β-CC), and linalool (LIN) for (A) VOC fumigation in a limited air volume and (B) VOC treatment as liquid spray.

| 1. VOC fumigation in a limited air volume | | | | |
| --- | --- | --- | --- | --- |
| VOC | **Concentration in a limited air volume  [mg L^-1^ air volume]** | **Amount of applied VOC [mL]** | **Amount of applied DMSO [mL]** | **Amount of deionized water [mL]** |
| 2-PE | 0.1 | 0.002 | 0.005 | 0.040 |
|  | 1 | 0.012 | 0.047 | 0.403 |
|  | 2 | 0.024 | 0.048 | 0.403 |
|  | 3 | 0.036 | 0.071 | 0.604 |
|  | 4 | 0.047 | 0.095 | 0.806 |
|  | 5 | 0.059 | 0.118 | 1.007 |
|  | 10 | 0.118 | 0.236 | 2.014 |
|  | 15 | 0.178 | 0.355 | 3.021 |
|  | 20 | 0.237 | 0.474 | 4.029 |
|  | 200 | 2.370 | 4.739 | 40.286 |
| β-CC | 0.1 | 0.002 | 0.003 | 0.026 |
|  | 1 | 0.015 | 0.030 | 0.257 |
|  | 2 | 0.030 | 0.060 | 0.514 |
|  | 3 | 0.045 | 0.091 | 0.771 |
|  | 4 | 0.060 | 0.121 | 1.028 |
|  | 5 | 0.071 | 0.141 | 1.199 |
|  | 10 | 0.141 | 0.282 | 2.399 |
|  | 15 | 0.212 | 0.423 | 3.598 |
|  | 20 | 0.302 | 0.604 | 5.138 |
|  | 200 | 3.022 | 6.045 | 51.379 |
| LIN | 1 | 0.014 | 0.028 | 0.242 |
|  | 2 | 0.028 | 0.057 | 0.483 |
|  | 3 | 0.043 | 0.085 | 0.725 |
|  | 4 | 0.057 | 0.114 | 0.967 |
|  | 5 | 0.071 | 0.142 | 1.209 |
|  | 10 | 0.142 | 0.284 | 2.417 |
| 1. VOC treatment as a liquid spray | | | | |
| VOC | **Concentration in liquid spray volume**  **[mg L^-1^ spray volume]** | **Amount of applied VOC [µL]** | **Amount of applied DMSO [µL]** | **Amount of deionized water [µL]** |
| 2-PE | 100 | 0.99 | 1.97 | 9,997.04 |
|  | 500 | 4.94 | 9.87 | 9,985.19 |
|  | 1,000 | 9.87 | 19.75 | 9,970.38 |
|  | 1,500 | 14.81 | 29.62 | 9,955.57 |
|  | 5,000 | 49.37 | 98.74 | 9,851.89 |
|  | 15,000 | 148.11 | 296.22 | 9,555.7 |
|  | 20,000 | 197.5 | 395.0 | 9,407.6 |
| β-CC | 100 | 1.26 | 2.52 | 9,996.22 |
|  | 500 | 6.30 | 12.59 | 9,981.11 |
|  | 1,000 | 12.59 | 25.19 | 9,962.22 |
|  | 1,500 | 18.89 | 37.78 | 9,943.33 |
|  | 5,000 | 62.96 | 125.93 | 9,811.18 |
|  | 15,000 | 188.89 | 377.37 | 9,433.32 |
|  | 20,000 | 251.9 | 503.7 | 9,244,4 |
| LIN | 100 | 1.18 | 2.37 | 9,996.45 |
|  | 500 | 5.92 | 11.85 | 9,982.23 |
|  | 1,000 | 11.85 | 23.70 | 9,964.45 |
|  | 1,500 | 17.77 | 35.55 | 9,946.68 |
|  | 5,000 | 59.25 | 118.50 | 9,822.25 |
|  | 15,000 | 177.75 | 355.49 | 9,466.8 |
|  | 20,000 | 237.0 | 474.0 | 9,289.0 |
