# Supplementary material for: Air volume not spray concentration determines in vivo efficacy of volatile organic compounds against Plasmopara viticola
Source: Sci Rep. 2026 Feb 16;16:9325. doi: 10.1038/s41598-026-40527-1 (PMC13000241; doi:10.1038/s41598-026-40527-1)
Supplement: Supplementary file 2 — Supplementary Material 2 [file 41598_2026_40527_MOESM2_ESM.docx]

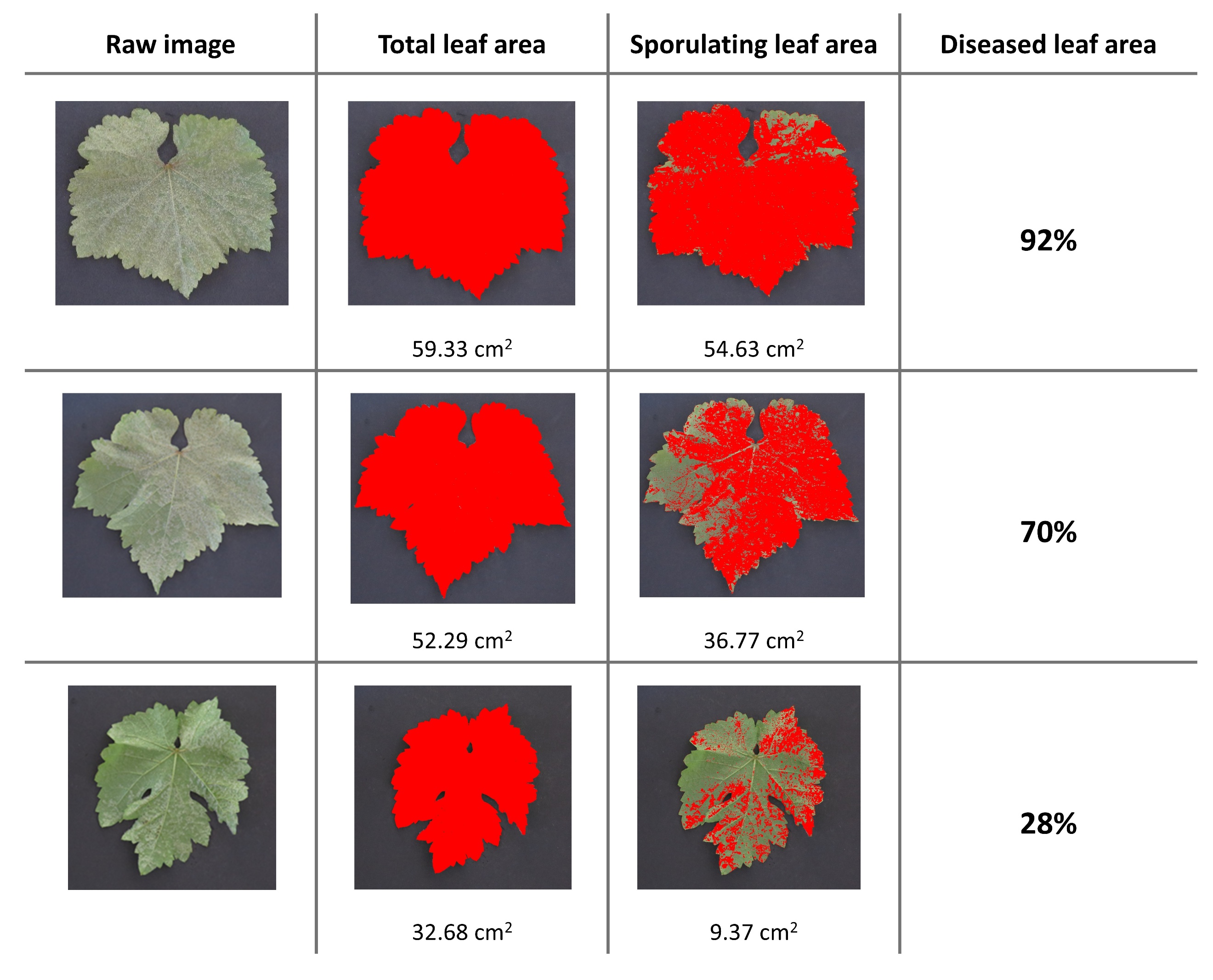


**Supplementary Figure 1:** The *Plasmopara viticola* diseased leaf area (in percentage) was quantified using Fiji software by applying different color thresholds to distinguish between total leaf area and sporulating leaf area. The captured leaf surface is highlighted in red and measured in cm².
